# Compilation of a Near-Infrared Library for Construction of Quantitative Models of Oral Dosage Forms for Amoxicillin and Potassium Clavulanate

**DOI:** 10.3389/fchem.2018.00184

**Published:** 2018-05-24

**Authors:** Wen-bo Zou, Xiao-meng Chong, Yan Wang, Chang-qin Hu

**Affiliations:** Antibiotic Division, National Institutes for Food and Drug Control, Beijing China

**Keywords:** near-infrared spectroscopy, universal model, sample selection, spectral library, quantitative analysis

## Abstract

The accuracy of quantitative models for near-infrared (NIR) spectroscopy is dependent upon calibration samples with concentration variations. Conventional sample-collection methods have shortcomings (especially time-consumption), which creates a “bottleneck” in the application of NIR models for Process Analytical Technology (PAT) control. We undertook a study to solve the problem of sample collection for construction of NIR quantitative models. Amoxicillin and potassium clavulanate oral dosage forms (ODFs) were used as examples. The aim of this study was to find an approach to construct NIR quantitative models rapidly using a NIR spectral library based on the idea of a universal model. The NIR spectral library of amoxicillin and potassium clavulanate ODFs was defined and comprised the spectra of 377 batches of samples produced by 26 domestic pharmaceutical companies, including tablets, dispersible tablets, chewable tablets, oral suspensions, and granules. The correlation coefficient (r_T_) was used to indicate the similarities of the spectra. The calibration sets of samples were selected from a spectral library according to the median r_T_ of the samples to be analyzed. The r_T_ of the samples selected was close to the median r_T_. The difference in r_T_ of these samples was 1.0–1.5%. We concluded that sample selection was not a problem when constructing NIR quantitative models using a spectral library compared with conventional methods of determining universal models. Sample spectra with a suitable concentration range in NIR models were collected rapidly. In addition, the models constructed through this method were targeted readily.

## Introduction

Near infrared spectroscopy (NIRS) is a rapid, low-cost, and non-destructive technology that has been used widely in quality control and for the rapid detection of pharmaceuticals (Jamrógiewicz, 2012; Chong et al., 2016; Dong et al., 2016). It has also been used to monitor pharmaceutical manufacturing online (Möltgen et al., 2012; Sarraguça et al., 2014; Wahl et al., 2014). In 2003, US The Food and Drug Administration (FDA) announced Pharmaceutical Current Good Manufacturing Practices (cGMPs) for the twenty-first century to obtain better knowledge of production processes. The document offers guidelines to secure pharmaceutical quality *via* process control of raw materials as well as intermediate and final products during manufacturing (Velagaleti et al., 2002; United States Food and Drug Administration, 2004). Process Analytical Technology (PAT) is the key point of process control during pharmaceutical production (United States Pharmacopeial Convention, 2015). NIRS is the most frequently used method of PAT because it is efficient, pollution-free and has no need for sample pretreatment (Hertrampf et al., 2015).

The accuracy of quantitative analysis depends on NIR models. Sample selection is challenged during the selection of NIR quantitative models. A sufficient number of samples are needed to comprise the appropriate concentration range necessary for the calibration set. However, collecting enough calibration samples with concentration variability in the PAT process is difficult.

Five methods have been proposed to collect calibration samples. The first method uses normal products and the development of samples, which are normally out of specification and can extend the concentration range (Gottfries et al., 1996; Merckle and Kovar, 1998; Corti et al., 1999). The second method uses standard additions for active pharmaceutical ingredients (APIs) or excipients to increase or decrease the sample concentration (Dreassi et al., 1996; Blanco et al., 1997, 2001). The third method uses laboratory-made samples by changing the concentration of the components in the matrix (Moffat et al., 2000; Blanco et al., 2001). The fourth method uses laboratory-made samples with production samples that comprise granules, tablet cores, and coated tablets (these are all sources of variation in the model) (Blanco et al., 1998). The fifth method uses a mixture of API and excipients in different proportions for preparation of laboratory-scale samples (Mafalda and Lopes, 2009).

These methods can broaden the range of the calibration concentration. However, the sample-preparation procedure is time-consuming. Also, the samples prepared in the laboratory are not “real” commercial products because they cannot encompass all the chemical and physical properties of commercial products (e.g., excipients, particle size, polymorphs). Besides, constructing models using underdosed and overdosed samples may carry problems in terms of the correlation between the concentrations of API and other excipients (Mafalda and Lopes, 2009). When constructing models of compound preparations, the underdosing/overdosing procedure should be done by means of a “sample concentration matrix.” This involves calculation of the cross-correlation between the constituents as their individual concentrations are increased or decreased, thereby avoiding spurious correlations among constituents (Blanco and Alcala, 2006). Therefore, sample selection remains a “bottleneck” in the application of NIR models for PAT control.

We have been studying NIR universal models (Feng et al., 2010). Such a universal model could be used to rapidly analyze pharmaceuticals from different manufacturers under the same international non-proprietary name (INN). A homologous sample based on the application of universal samples has been proposed (Zou et al., 2013). A set of samples are considered “homologous” if they contain the same API, similar excipients, and similar production processes. The NIR spectra of the samples in one homologous sample set are, therefore, highly similar. Calibration sets in the universal model comprise several homologous samples. Samples can be accurately analyzed *via* universal models if they fall into homologous samples from the calibration set. Errors may occur, and the original model should be updated if the universal model analyzes a new sample that cannot be covered by the existing homologous sample sets. Universal models do not need sample preparation. All of the calibration and validation samples can be obtained in the market. The method of sample selection ensures an appropriate range of calibration concentration, which is important to develop a robust calibration.

Amoxicillin and potassium clavulanate are compound preparations of β-lactam and β-lactamase inhibitors, respectively. They are used for the treatment of bacterial infections of the respiratory and urinary tracts. The oral dosage forms (ODFs) for amoxicillin and potassium clavulanate combined in different ratios are tablets (7:1, 4:1, 2:1), dispersible tablets (14:1, 7:1, 4:1), chewable tablets (8:1, 2:1), granules (7:1, 4:1), and oral suspensions (7:1, 4:1, 2:1). Universal models of tablets of amoxicillin and potassium clavulanate are constructed to measure the content of amoxicillin, potassium clavulanate, water, and the major impurity: cycle-closed dimer (Chong et al., 2016). Some NIR methods have been proposed for determination of amoxicillin in suspensions and capsules, in which calibration samples are formulated similar to those for commercial products (Silva et al., 2012; Khan et al., 2016).

Herein, we took the concept of a universal model to build a NIR spectral library of ODFs for amoxicillin and potassium clavulanate by collecting various products with different strengths from different manufacturers. Calibration samples could be chosen from the NIR spectral library when establishing NIR universal models to determine the contents of amoxicillin, potassium clavulanate, and/or water in the PAT control. Samples were considered to be homologous if they were similar to calibration samples. The feasibility of constructing NIR models using a NIR spectral library was discussed. Thus, the problem of collecting calibration samples could be resolved by PAT control.

## Materials and methods

### Samples and reagents

Three hundred and seventy seven batches of amoxicillin and potassium clavulanate ODFs produced by 26 manufacturers were collected in post-marketing surveillance in 2012 and 2014. There were 74 batches of tablets, 78 batches of dispersible tablets, 10 batches of chewable tablets, 96 batches of granules, and 120 batches of oral suspensions; 211 samples of amoxicillin capsule were from 100 batches provided by ZhuHai United Laboratories. The amoxicillin capsules included mixed intermediate granules of amoxicillin capsules as well as filled capsules and/or packaged capsules of the same batch. A reference standard of amoxicillin trihydrate (lot number: 130409-201011; content: 85.8%) and potassium clavulanate (lot number: 130429-201307; content: 95.0%) were provided by the US National Institutes for Food and Drug Control.

Methanol was purchased from Fisher Scientific (Pittsburgh, PA, USA). Phosphoric acid was obtained from Beijing Chemical Works (Beijing, China). Sodium dihydrogen phosphate dihydrate was purchased from Sinopharm Chemical Reagents (Beijing, China).

### Reference method

The reference contents of amoxicillin and potassium clavulanate were determined by high-performance liquid chromatography (HPLC) (Chong et al., 2016) using an Ultimate 3000 HPLC system (Dionex, Sunnyvale, CA, USA) and an ZORBAX SB-C18 column (5 μm, 150 × 4.6 mm; Agilent Technologies, Santa Clara, CA, USA). The chromatographic conditions were: column temperature, 30°C; detection wavelength, 220 nm; flow rate, 1 mL min^−1^; injection volume, 20 μL; mobile phase, 5:95 (v/v) methanol/phosphate buffer (0.05 mol L^−1^ sodium dihydrogen phosphate pH adjusted to 4.4 with 10% phosphoric acid).

For each tablet or granule/oral suspension of amoxicillin and potassium clavulanate, 10 tablets or 10 bags of granules/oral suspensions were pulverized in a motor, weighed accurately, dissolved in the mobile phase to get 0.5 mg mL^−1^ of amoxicillin or potassium clavulanate for HPLC analysis. Two replicate runs were done for each sample to get the average reference value. The water content was determined *via* the Karl Fischer method according to the *Chinese Pharmacopoeia*.^1^

### Acquisition and pre-processing of NIR spectra

Acquisition of NIR spectra was done on a MATRIX-F FT-NIR spectrometer (Bruker Optics, Billerica, MA, USA) equipped with a 1.5-mm fiberoptic diffuse reflectance probe and an extended TE-cooled indium gallium arsenide (InGaAs) detector. Data were collected and processed using OPUS v6.5 software (Bruker Optics).

The fiberoptic probe was used to record diffuse reflectance spectra at 8 cm^−1^ resolution in the spectral range 4,000–12,000 cm^−1^. During each measurement, 32 co-added scans were undertaken. The measurement was carried out by putting the fiberoptic diffuse reflectance probe close to the sample. For each tablet, dispersible tablet, and chewable tablet of amoxicillin and potassium clavulanate, three tablets were selected randomly and measured. The weight of each tablet was 0.5–1.0 g. Three sample bags, weighing 3.0–6.0 g, were selected randomly and measured for a granule and oral suspension of amoxicillin and potassium clavulanate. For each mixed intermediate granule of an amoxicillin capsule, 5 g of powder was placed in a vial and measured in triplicate. For each filled capsule and packaged capsule of amoxicillin, 5 g of powder of the capsule was placed in a vial and measured thrice. The three original spectra were averaged by OPUS v6.5 software. The average spectra were then subjected to a Savitzky–Golay first derivative treatment with 17-point smoothing, followed by vector normalization transformation. The pre-processed spectra were used for construction and validation of the model.

### Compilation of a NIR spectral library

The NIR spectral library comprised the spectra of 377 batches of amoxicillin and potassium clavulanate ODFs produced by 26 manufacturers (74 batches of tablets, 78 batches of dispersible tablets, 10 batches of chewable tablets, 96 batches of granules, and 120 batches of oral suspensions). For the NIR spectra of the library, the content of amoxicillin was 4.77–57.86%, the content of potassium clavulanate was 1.03–20.17%, and the water content was 0.24–9.30%. The correlation coefficient r_T_ between the spectra of each amoxicillin and potassium clavulanate ODF in the library and average spectra of tablets of amoxicillin and potassium clavulanate were calculated from 4,200 to 10,000 cm^−1^. The r_T_ ranged from 34.42 to 99.69% with an average of 71.78%. The r_T_ (Equation 1) of the two spectra y_1_ (k) and y_2_ (k) was calculated as the ratio of their covariance to the product of the two standard deviations σ_y1_ and σ_y2_. The value of r_T_ ranges from −1 (inverted spectra) to +1 (identical spectra) and is expressed as a percentage.

(1) $${\text{r}}_{\text{T}}=\frac{Cov(y_{1}(k),y_{2}(k))}{\sigma_{y_{1}}\sigma_{y_{2}}}$$

### Construction of the NIR quantitative model

Calibration models were constructed using the PLS1 algorithm (PLS regression for one *y*-variable) (Brereton, 2000; Burns and Ciurczak, 2008) available in the Quant 2 package of OPUS v6.5 software. The Rank value is the number of main factors in building the PLS model. Validation methods of calibration model include a Test Set Validation (TSV) and Leave-One-Out Cross Validation (LOOCV). In the relevant Figures and Tables, rank is the number of PLS latent variables (LV), which is determined by a one-sided *F*-test on PRESS (Equation 2). *R*^2^ (Equation 3) is the coefficient of determination, and gives the percentage of variance present in the true component values, which is reproduced in the prediction. M is the number of samples of the validation set. Y_m_ is the mean of true concentration values. Differ_i_ (Equation 4) is the difference between the true value and predicted value. RMSEP (Equation 5) is the root-mean-standard error of prediction in TSV. RMSECV (Equation 6) is the root-mean-standard error of LOOCV. Principal Component Analysis (PCA) scores indicate the position (coordinates) of the samples. PCA is calculated on the basis of calibration spectra.

(2) $$\text{PRESS}=\sum_{\text{i}=1}^{\text{M}}{\left({\text{Differ}}_{\text{i}}\right)}^{2}$$

(3) $${\text{R}}^{\text{2}}=\left(\text{1}-\frac{\sum_{\text{i}=\text{1}}^{\text{M}}{\left({\text{Differ}}_{\text{i}}\right)}^{\text{2}}}{\sum_{\text{i}=\text{1}}^{\text{M}}{\left({\text{Y}}_{\text{i}}-{\text{Y}}_{\text{m}}\right)}^{\text{2}}}\right)\times \text{100}$$

(4) $${\text{Differ}}_{\text{i}}={\text{Y}}_{\text{i}}{\;}^{\text{true}}-{\text{Y}}_{\text{i}}{\;}^{\text{pred}}$$

(5) $$\text{RMSEP}=\sqrt{\frac{\text{1}}{{\text{M}}_{\text{t}}}\cdot \sum_{\text{i}=\text{1}}^{{\text{M}}_{\text{t}}}{\left({\text{Differ}}_{\text{i}}\right)}^{\text{2}}}$$

(6) $$\text{RMSECV}=\sqrt{\frac{\text{1}}{{\text{M}}_{\text{l}}}\cdot \sum_{\text{i}=\text{1}}^{{\text{M}}_{\text{l}}}{\left({\text{Differ}}_{\text{i}}\right)}^{\text{2}}}$$

#### Conventional method of construction of a universal quantitative model

A universal model was constructed based on our reported method (Chong et al., 2016). That is, all sample spectra were grouped into hierarchical clusters based on the Euclidean distance calculated from the Ward algorithm, and 19 groups were set according to the sample-selection strategy (Jia et al., 2011). Three random samples from each cluster were selected. Two of these samples were composed of the calibration set, and the remaining one was the validation set. Sixty-four spectra were selected to establish the NIR quantitative model to analyze the content of amoxicillin, potassium clavulanate, and water. Some test spectra for which the prediction differences were greater than the expected values, were transferred to the calibration set to optimize the model.

#### Construction of a universal quantitative model using the NIR spectral library

All spectra were sequenced *via* the r_T_ value. One spectrum was selected according to differences in r_T_ values to construct a NIR quantitative model. Two-thirds of these spectra were composed of the calibration set; whereas one-third of the spectra were in the test set. Some spectra from the test set, for which the prediction difference was greater than expected, were transferred to the calibration set to optimize the model. These spectra could be used to analyze the content of amoxicillin, potassium clavulanate, and water.

#### Validation of the accuracy of the NIR quantitative model

The accuracy of NIR quantitative models was evaluated by Prediction Difference, which was the difference between the predicted content and reference content of amoxicillin, clavulanate, and water.

Prediction Difference = |Prediction Content − Reference Content|

### Sample measurements

Here, 377 batches of amoxicillin and potassium clavulanate ODFs from post-marketing surveillance were measured in two time periods: 137 samples were measured for about 3 months in 2012, and the others were measured for about 6 months in 2014.

Spectra of 211 amoxicillin capsule samples were acquired for PAT control at ZhuHai United Laboratories (Guangdong Sheng, China) for about 7 months in 2016.

## Experimental design

**Figure 1:**
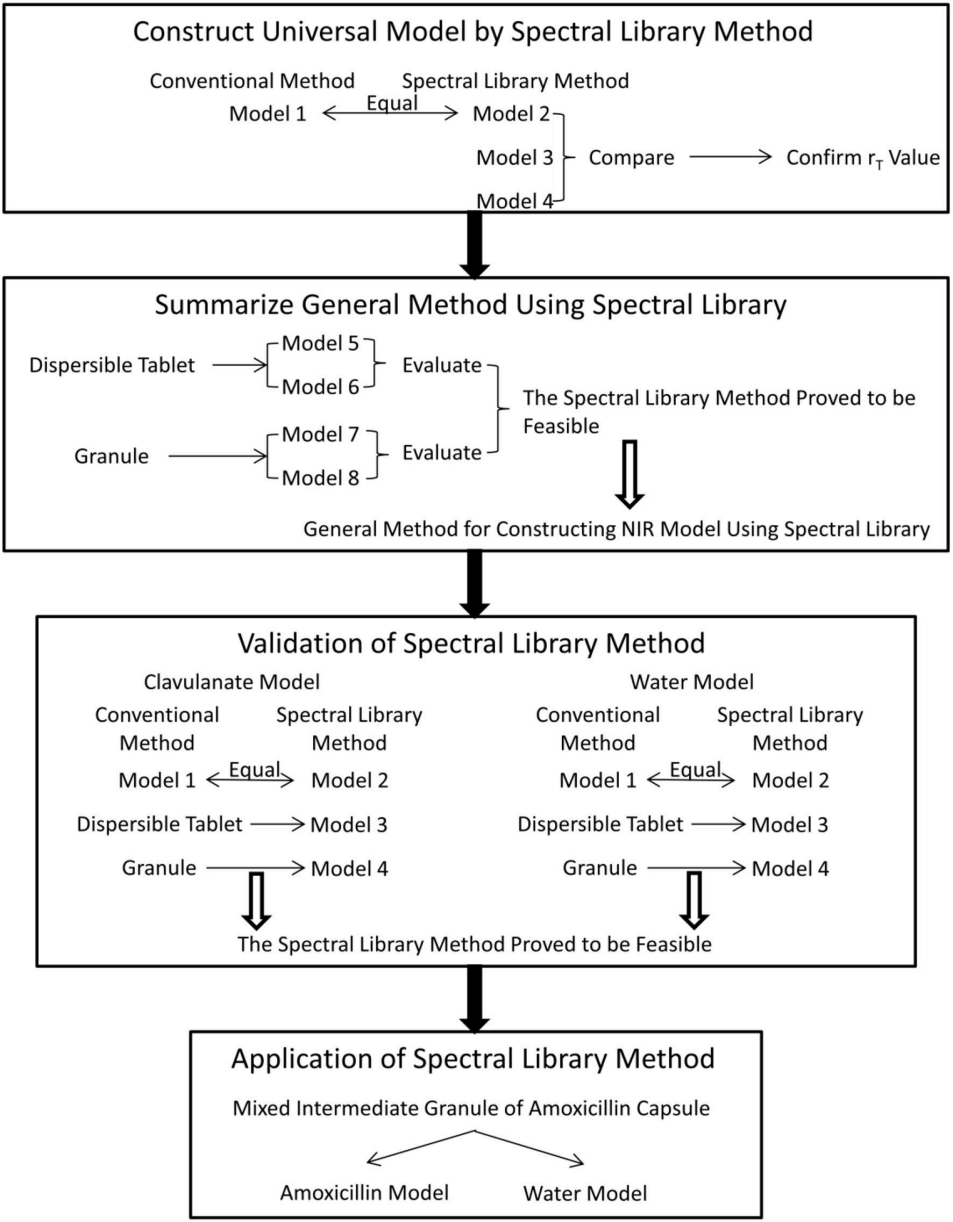
Experimental design and list of all models.

Four steps were designed in the experiment (Figure 1). At first, a universal model (model 1) of amoxicillin for all amoxicillin and potassium clavulanate ODFs was constructed using a conventional calibration method for sample selection. Then, the NIR spectral library was used for modeling (model 2). Model 1 was used as a reference model to compare with model 2. If the results analyzed by model 2 were close to those analyzed by model 1, the spectral library was effective. Simultaneously, the appropriate difference between r_T_ values of adjacent spectra in the calibration set was tested (models 2, 3, and 4). At the second step, models for a dispersible tablet (models 5 and 6) and models for a granule (models 7 and 8) were constructed using a general method for constructing a NIR model using spectral library. If the general models performed well, it was validated by constructing models for analyzing potassium clavulanate (clavulanate models 1, 2, 3, and 4) and water content (water models 1, 2, 3, and 4) in the third step. Finally, the spectral-library method was applied to a real PAT control. Models for analyzing amoxicillin and water content in mixed intermediate granules of amoxicillin capsules were constructed.

## Results and discussion

**Figure 2:**
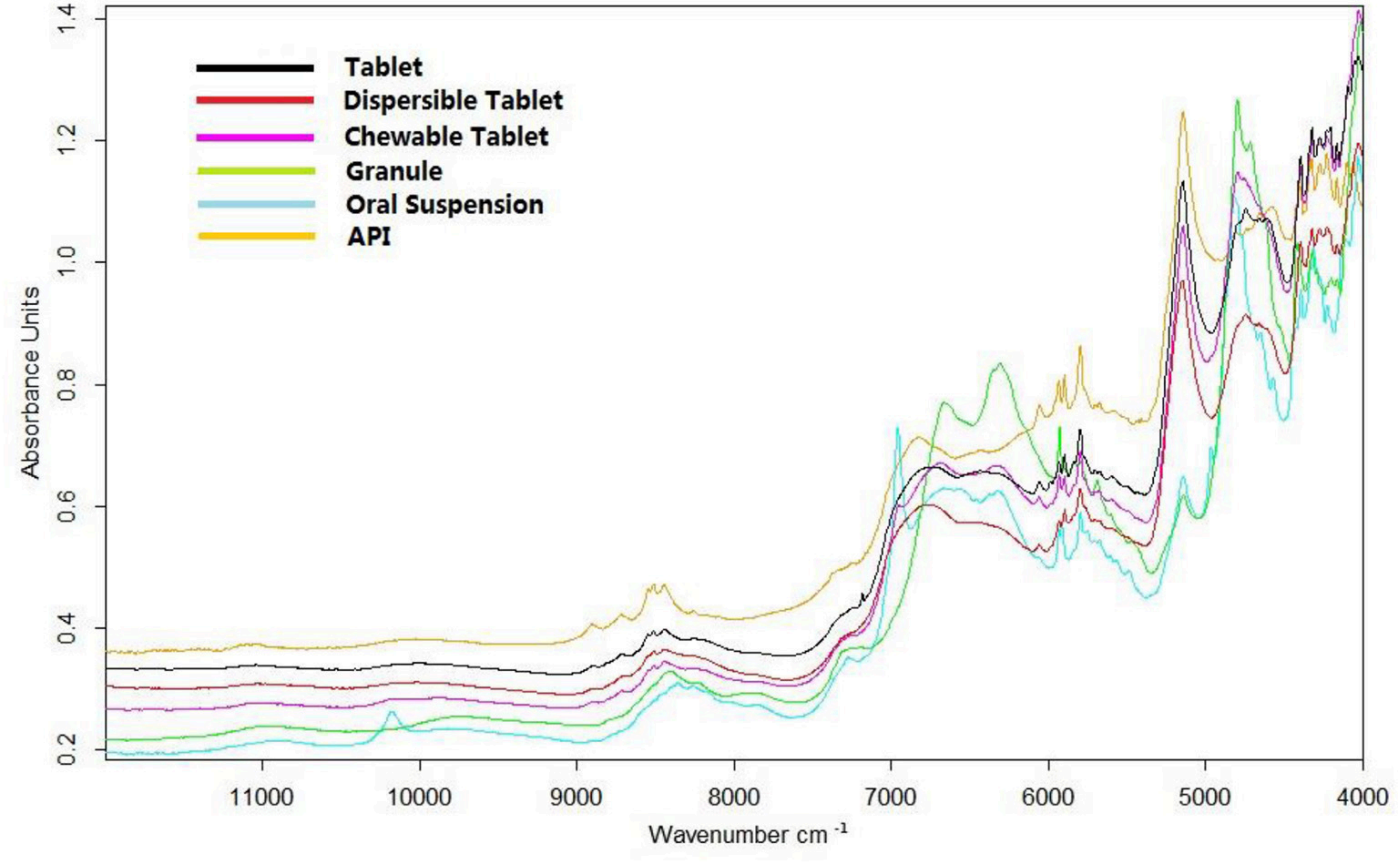
Representative spectra of a tablet, dispersible tablet, chewable tablet, granule, and oral suspension of amoxicillin and potassium clavulanate, and the spectrum of amoxicillin.

Representative spectra of a tablet, dispersible tablet, chewable tablet, granule, and oral suspension of amoxicillin and potassium clavulanate are shown in Figure 2. The spectra of a tablet, dispersible tablet, and chewable tablet are similar. Due to their prescription, low strength, and production process, the spectra of granule and oral suspension are quite different from those of a tablet, dispersible tablet, and chewable tablet. The spectrum of the amoxicillin API (Figure 2) was similar to the spectra of amoxicillin and potassium clavulanate ODFs in some spectral regions, such as the bands between 8,300 and 9,500 cm^−1^ (overtone of C-H stretching vibrations), and between 5,300 and 6,500 cm^−1^, 4,200 and 4,800 cm^−1^ (overtone of C = O bonds). The calibration models analyzing amoxicillin could be set up on the basis of these spectral ranges.

### Universal quantitative model for amoxicillin ODFs

**Figure 3:**
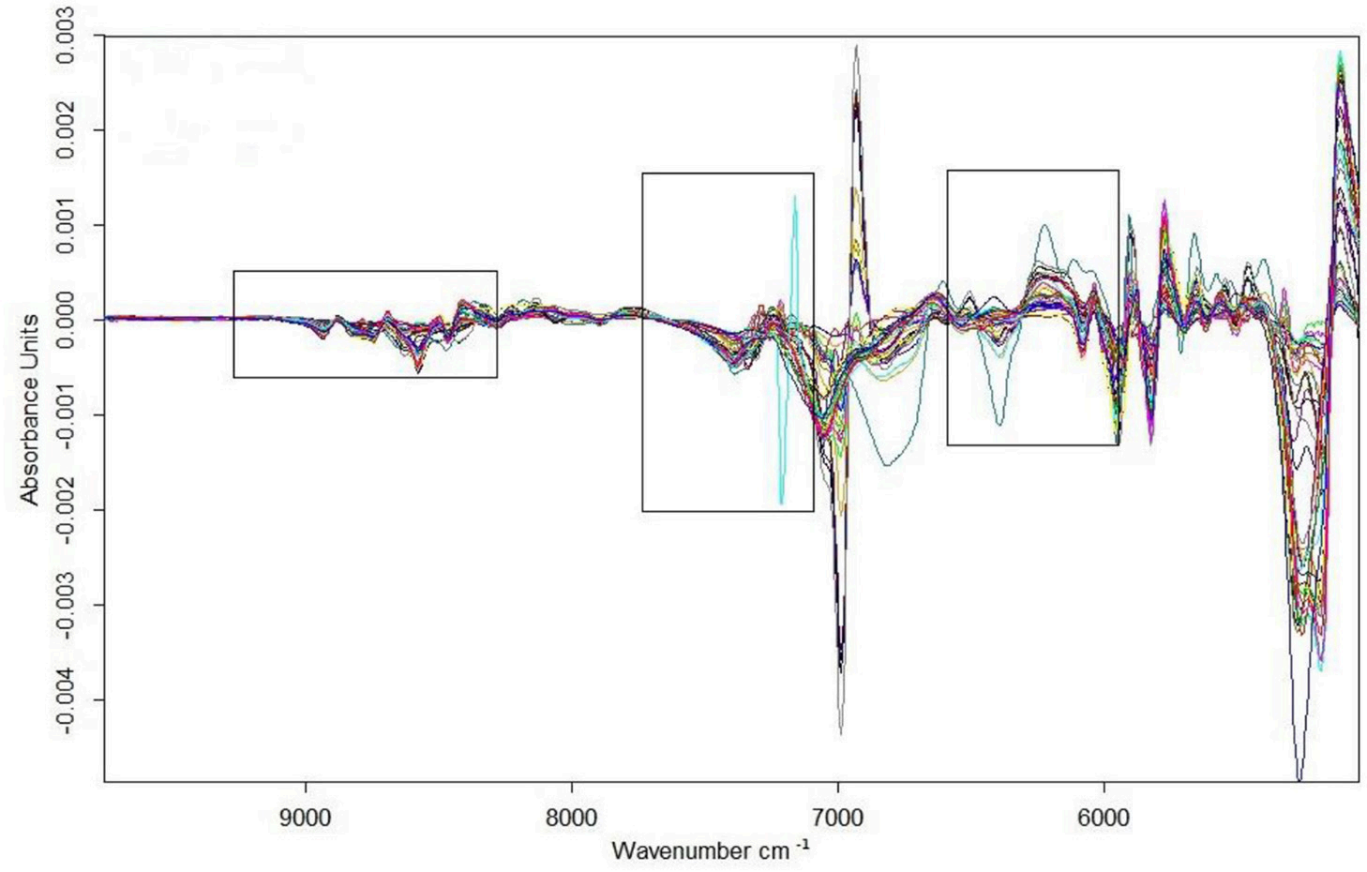
Spectra of calibration samples of model 1 after being first-derivative preprocessed with a modeling spectral region.

**Figure 4:**
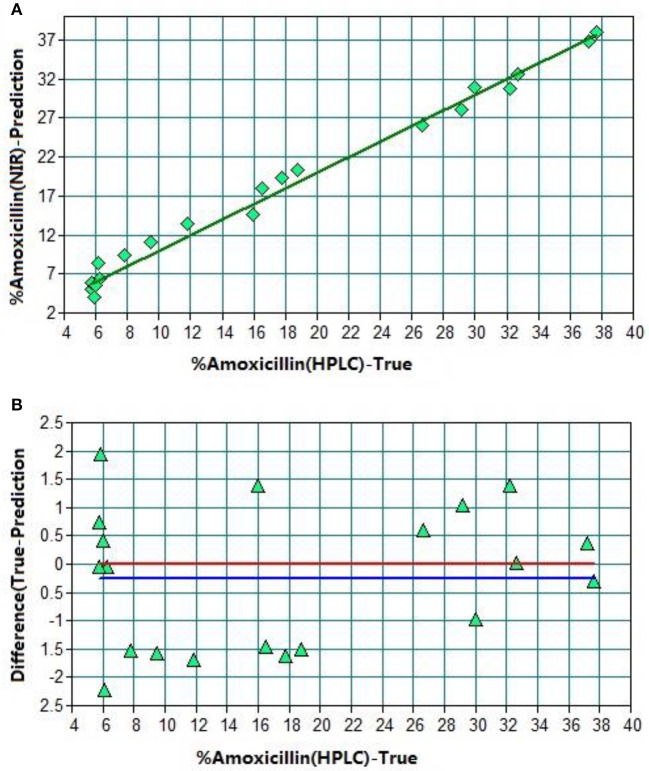
TSV of model 1. **(A)** Plot of prediction values vs. true values. **(B)** Plot of difference vs. true values. The red line shows the zero line of difference. The blue line shows deviation of the zero line.

The universal quantitative model for amoxicillin set up using the conventional method was called “amoxicillin model 1” (model 1). The spectral range employed for model 1 is shown in Figure 3. Figure 4 shows the result of test-set validation of model 1.

After optimization, there were 44 sample spectra for the calibration set (training set) and 20 for the validation set (test set). *R*^2^ was found to be 98.83% with RMSEP 1.23% (Table 1). The average predicted difference between the predicted content and reference content of amoxicillin was only 1.3%. Hence, this NIR method could be a replacement of the HPLC method.

**Table 1:** Parameters and prediction differences of models 1, 2, 3, and 4.

**Parameter**	**Model 1**		**Model 2**		**Model 3**		**Model 4**	

	**Training set**	**Test set**	**Training set**	**Test set**	**Training set**	**Test set**	**Training set**	**Test set**
Number of samples	44	20	46	19	25	8	s61	25
Content range (%, mg/mg)	5.78–39.20	5.88–37.65	5.17–53.56	5.23–39.65	4.85–57.86	5.87–29.31	4.85–57.86	5.45–53.33
Wavenumber range (cm^−1^)	9426.6–8273.4, 7702.5–7124.0, 6549.3–5970.7		9426.6–7124.0, 6549.3–5396.0, 4825.2–4246.6		7702.5–7124.0, 6549.3–5396.0, 4825.2–4246.6		9426.6–7124.0, 6549.3–5396.0, 4825.2–4246.6	
Pre-processing method	1st derivative + vector normalization		1st derivative + vector normalization		1st derivative + vector normalization		1st derivative + vector normalization	
Rank	5		5		4		4	
*R*^2^ (%)	98.83		99.13		99.57		99.00	
RMSEP (%)	1.23		1.25		0.509		1.29	
r_T_ Difference (%)	1.5		1.0		2.0		0.75	
Samples whose prediction difference is >5%	3.7% (14/377)		3.7% (14/377)		7.4% (28/377)		5.0% (19/377)	
Samples whose prediction difference is <1%	46.2% (174/377)		43.0% (162/377)		29.0% (109/377)		40.0% (151/377)	
Average prediction difference	1.3%		1.6%		2.0%		1.7%	

The universal quantitative model for amoxicillin constructed using the NIR spectral library was called “amoxicillin model 2” (model 2). Model 2 and the subsequent models were optimized and validated by the same method as that used for model 1. The difference in r_T_ values between adjacent spectra using model 2 was about 1.0%. The results of the two models were close (Table 1), so they had the same analysis capacity for amoxicillin.

The average of the difference between r_T_ values of adjacent spectra in the calibration set of model 1 was about 1.5%. The influence of the difference between r_T_ values of adjacent spectra was also investigated. “Amoxicillin model 3” (model 3) and “amoxicillin model” 4 (model 4) were constructed with a difference of 2.0 and 0.8%, respectively. The prediction differences of 377 samples analyzed by models 1, 2, 3, and 4 were compared (Table 1). We found that the prediction differences of models 1, 2, and 4 were close and less than that of model 3—especially with samples, whose deviation was >5%. When there were large differences between r_T_ values of adjacent spectra, the calibration samples decreased and became less representative. A difference of 1.5% was suitable for modeling.

### Construction of NIR quantitative models for specific ODFs of amoxicillin using a spectral library

We used dispersible tablets of amoxicillin and potassium clavulanate as an example to establish a universal quantitative model for one dosage form (Table 2). Calibration sample spectra were selected according to the r_T_ value from a spectral library comprising 377 spectra of amoxicillin and potassium clavulanate ODFs. At first, only the calibration spectra of dispersible tablets were selected from the spectral library. The 78 spectra of dispersible tablets were sequenced by r_T_ value, and 30 spectra were chosen with a difference between r_T_ values of adjacent spectra of 1.0–1.5% to construct “amoxicillin model” 5 (model 5).

**Table 2:** Parameters of models 5, 6, 7, and 8.

**Parameter**	**Model 5**	**Model 6**	**Model 7**	**Model 8**
	**Training set**	**Training set**	**Training set**	**Training set**
Number of samples	30	30	30	30
Content range (%, mg/mg)	18.57–40.32	7.97–57.86	4.85–8.05	4.77–22.27
Wavenumber range (cm^−1^)	8277.2–7698.7, 5399.9–4821.2	6549.3–5970.7, 4825.2–4246.6	5797.2–5276.5	8851.9–8273.4, 6549.3–5970.7
Pre-processing method	1st derivative + vector normalization	1st derivative + vector normalization	1st derivative + vector normalization	1st derivative + vector normalization
Rank	4	4	5	5
*R*^2^ (%)	97.48	96.46	96.28	94.07
RMSECV (%)	1.14	2.32	0.197	1.20

**Figure 5:**
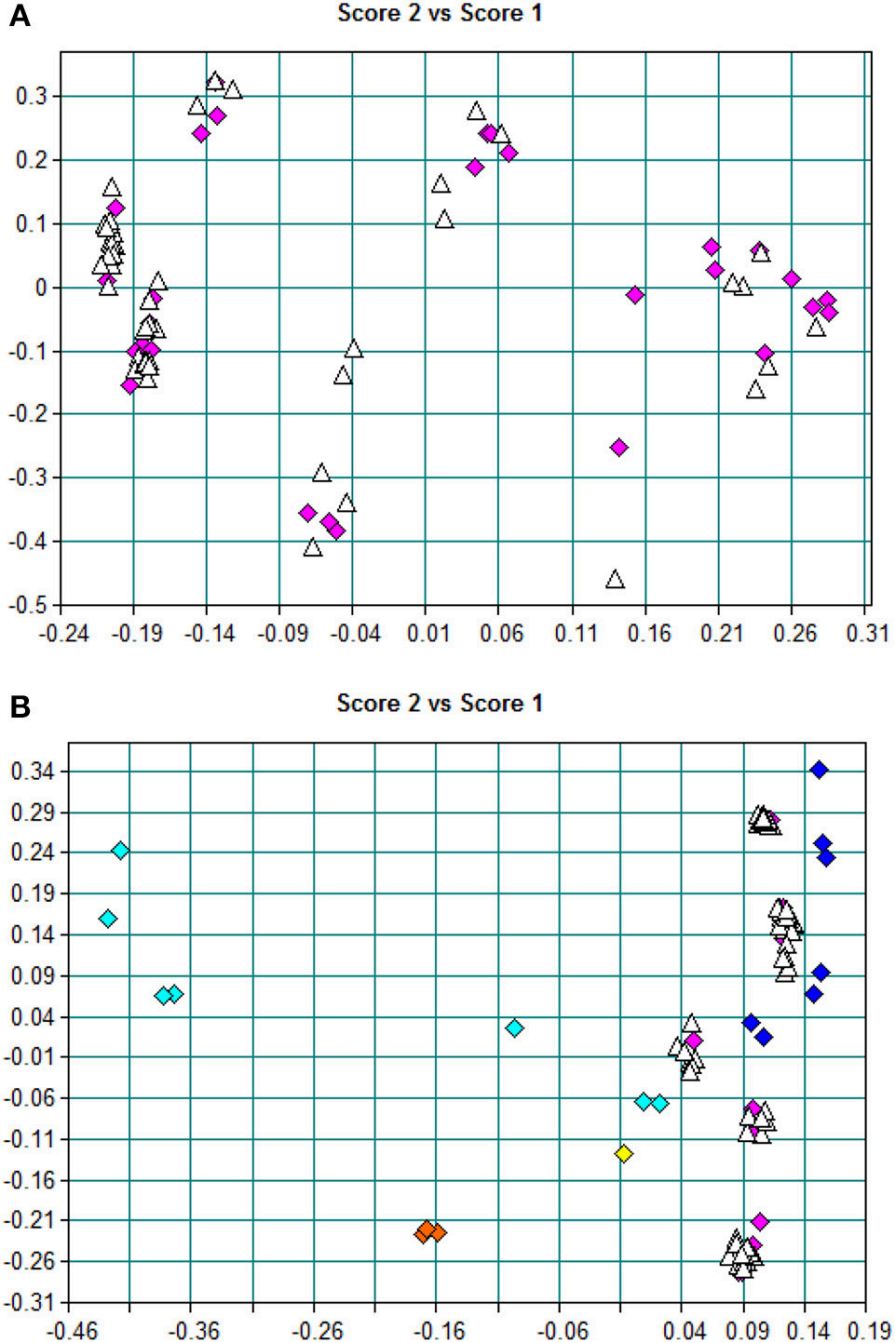
**(A)** PCA scores of model 5; pink blocks represent training-set spectra, and white triangles represent other spectra of dispersible tablets in the spectral library. **(B)** PCA scores of model 6; blocks represent training-set spectra, dark-blue blocks represent tablets, pink blocks represent dispersible tablets, yellow blocks represent chewable tablets, light-blue blocks represent oral suspensions, orange blocks represent granules, and white triangles represent other spectra of dispersible tablets in the spectral library.

“Amoxicillin model 6” (model 6) was established in a similar way. It means that the spectra of all dosage forms were selected for calibration. The average r_T_ value of 78 dispersible tablets was nearly the median value of r_T_ of the 30 calibration spectra, among which there were 12 spectra for a dispersible tablet. Comparing the PCA-score distribution space of models 5 and 6, the calibration samples of model 5 covered almost all of the distribution space of dispersible tablets; whereas the calibration samples of model 6 covered more space than model 5 (Figure 5). The prediction results of 78 batches of dispersible tablets by models 1, 2, 5, and 6 are shown in Table 3. The prediction differences seen in models 5 and 6 were lower than in the other two models. It is clearly indicated that it was feasible to construct NIR quantitative models of dispersible tablets of amoxicillin and potassium clavulanate using a spectral library.

**Table 3:** Predictions of dispersible tablets and granules of amoxicillin and potassium clavulanate by amoxicillin models.

**Model**	**r_*T*_ Difference**	**Dosage form**	**Number of samples**	**Samples whose prediction difference is >5%**	**Samples whose prediction difference is <1%**	**Average prediction difference**
1	1.5%	Dispersible tablets	78	2.6%	53.8%	1.3%
		Granules	96	0%	53.1%	1.0%
2	1.0%	Dispersible tablets	78	6.4%	43.6%	1.9%
		Granules	96	0%	51.0%	1.2%
5	0.7%	Dispersible tablets	78	0%	64.1%	0.8%
6	0.9%	Dispersible tablets	78	0%	52.6%	1.2%
7	1.6%	Granules	96	0%	100.0%	0.1%
8	1.1%	Granules	96	0%	81.2%	0.8%

The prescription and production process of tablets/dispersible tablets and granules/oral suspensions are quite different. As a result, the spectra of those dosage forms differed greatly (Figure 2). On this occasion, granules were taken as an example to validate the feasibility of constructing NIR quantitative models using a spectral library.

Models 7 and 8 were established similar to models 5 and 6. Thirty spectra were selected with a difference between r_T_ values of adjacent spectra of 1.0–1.5%. Calibration spectra from model 7 were chosen from 96 spectra of granules. Samples from model 8 were from all dosage forms in the spectral library. Because the r_T_ value of the oral suspension was close to that of a granule, 13 oral suspension spectra were comprised by model 8. A tablet spectrum was not included in model 8. The prediction values of all the granules included by the spectral library by models 1, 2, 7, and 8 are shown in Table 3. The results of models 7 and 8 were better.

Dispersible tablets of amoxicillin and potassium clavulanate could be analyzed equally well by models 5 and 6. Similar results could be obtained for granules by models 7 and 8. The r_T_ value was critical for sample selection, but it was not necessary to choose the same dosage form as the samples to be measured.

**Figure 6:**
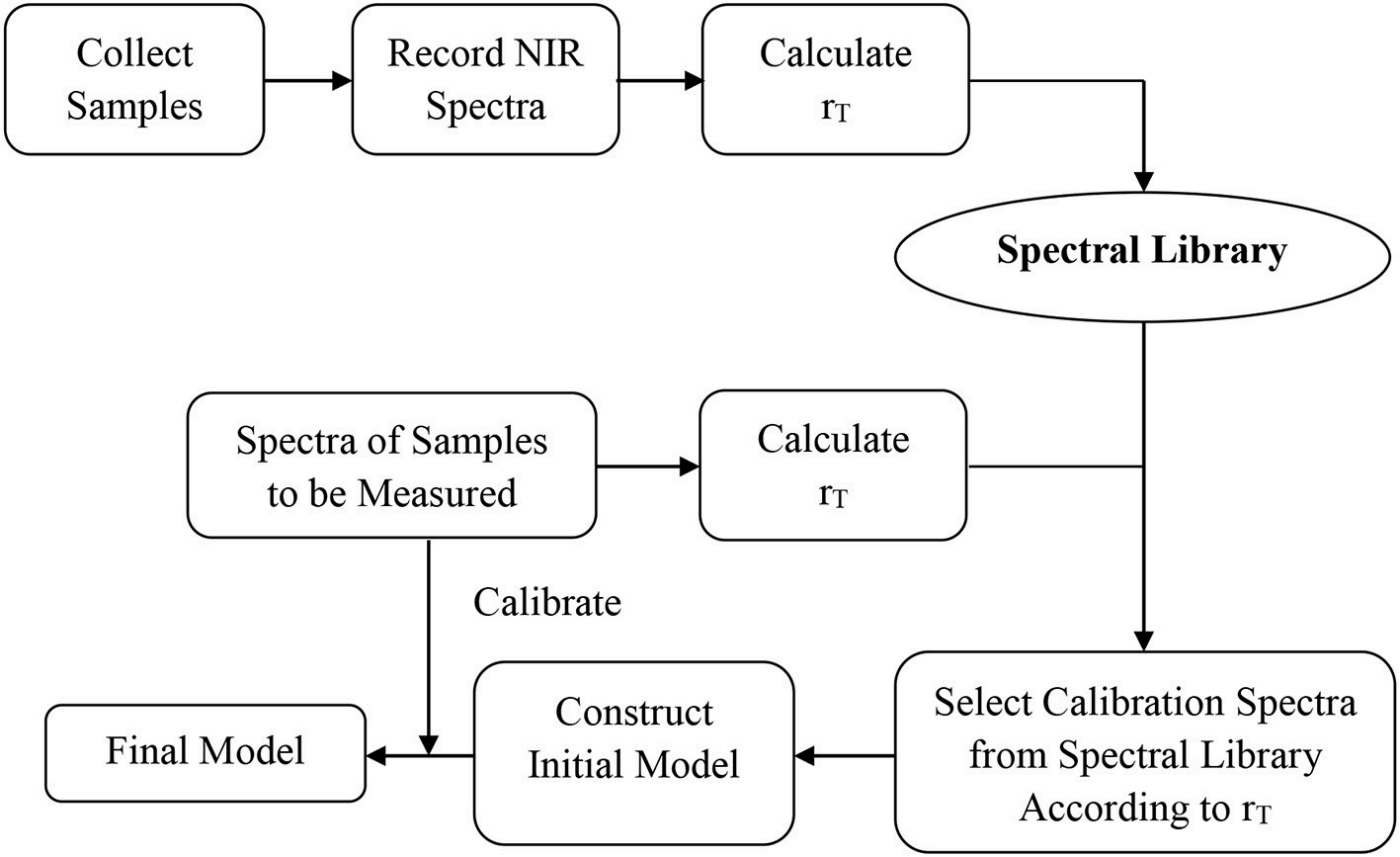
General method for constructing a NIR model using a spectral library.

A general method for constructing NIR quantitative models using a spectral library was summarized based on the experiments above (Figure 6). Firstly, the appropriate spectra of the samples to be measured were acquired, and the r_T_ value calculated according to the definition of r_T_ in the spectral library. Secondly, the calibration samples were selected based on the median r_T_ value of samples to be measured. The difference between r_T_ values of adjacent spectra in the calibration set was about 1.0–1.5%. The number of calibration samples should be ≥ 30, and their r_T_ value should cover the range of samples to be measured. Finally, the model accuracy is validated by the samples to be measured. Appropriate sample spectra could be added to the calibration set to optimize the model if necessary.

### Validation of the general method for constructing a NIR quantitative model using a spectral library

#### Constructing NIR quantitative models for potassium clavulanate

A universal quantitative model for potassium clavulanate (“clavulanate model 1”) was set up as shown in section Conventional Method of Construction of a Universal Quantitative Model. “Clavulanate model 2” was constructed by the general method as shown in Figure 6. The parameters and prediction difference for 377 samples in the spectral library of the two models (Table 4) indicated that the two methods of constructing models could lead to ideal results.

**Table 4:** Parameters and prediction differences of clavulanate models.

**Parameter**	**Clavulanate Model 1**		**Clavulanate Model 2**		**Clavulanate Model 3**		**Clavulanate Model 4**	

	**Training set**	**Test set**	**Training set**	**Test set**	**Training set**	**Test set**	**Training set**	**Test set**
Number of samples	53	16	46	19	30	12	32	10
Content range (%, mg/mg)	1.13–17.62	1.40–15.79	1.13–20.12	5.18–17.61	1.10–19.60	1.16–14.63	1.03–3.14	1.29–3.01
Wavenumber range (cm^−1^)	7702.5–7124.0,6549.3–5970.7		10001.3–8848.1, 6549.3–5396.0		6549.3–5970.7,4825.2–4246.6		10001.3–8848.1,6549.3–5396.0	
Pre-processing method	1st derivative + vector normalization		1st derivative + vector normalization		1st derivative + vector normalization		1st derivative + vector normalization	
Rank	5		6		6		6	
*R*^2^ (%)	99.63		99.48		99.63		99.78	
RMSEP (%)	0.271		0.386		0.226		0.0317	
Samples whose prediction difference is >5%	1.8% (7/377)		1.8% (7/377)		0% (0/78)		0% (0/96)	
Samples whose prediction difference is <1%	69.0% (260/377)		72.1% (272/377)		60.0% (47/78)		93.7% (90/96)	
Average prediction difference	0.8%		0.9%		0.8%		0.2%	

Similar to section Universal Quantitative Model for Amoxicillin ODFs, clavulanate model 3 (for dispersible tablets) and clavulanate model 4 (for granules) were constructed by the general method mentioned above. The prediction difference of 78 batches of dispersible tablets and 96 batches of granules by clavulanate models 3 and 4 were both < 1.0% (Table 4). These two models were accurate and reliable. The feasibility of establishing a NIR quantitative model using the spectral library was also demonstrated.

#### Constructing NIR quantitative models for water content

Universal quantitative models for water content (water model 1, 2, 3, 4) were established as shown in section Conventional Method of Construction of a Universal Quantitative Model, and the general method for a spectral library consequently resulted as shown in section Constructing NIR Quantitative Models for Potassium Clavulanate (Table 5). Water models 1 and 2 could be used to analyze all the dosage forms of amoxicillin and potassium clavulanate in the spectral library. Water models 3 and 4 could be used to analyze dispersible tablets and granules, respectively. Table 5 shows that the prediction differences of the four models was <1.0%. These data further validated the validity of the general modeling method using a spectral library.

**Table 5:** Parameters and prediction differences of water models.

**Parameter**	**Water Model 1**		**Water Model 2**		**Water Model 3**		**Water Model 4**	

	**Training set**	**Test set**	**Training set**	**Test set**	**Training set**	**Test set**	**Training set**	**Test set**
Number of samples	47	19	46	16	30	10	32	11
Content range (%, mg/mg)	1.39–9.29	1.50–7.17	0.41–8.98	1.29–7.67	1.42–8.73	1.89–7.08	0.50–2.60	1.30–2.28
Wavenumber range (cm^−1^)	9403.5–7498.1		10502.8–9951.2, 7752.7–5546.4		10502.8–8848.1, 8304.2–7197.3		8851.9–7748.8, 6101.9–5546.4	
Pre-processing method	1st derivative + vector normalization		1st derivative + vector normalization		1st derivative + vector normalization		1st derivative + vector normalization	
Rank	3		5		6		2	
*R*^2^ (%)	99.33		99.67		99.23		93.59	
RMSEP (%)	0.174		0.117		0.13		0.0737	
Samples whose prediction difference is >5%	0% (0/377)		0% (0/377)		0% (0/78)		0% (0/96)	
Samples whose prediction difference is <1%	87.0% (328/377)		80.9% (305/377)		47.4% (37/78)		96.9% (93/96)	
Average prediction difference	0.4%		0.5%		1.0%		0.4%	

### Application of the method for constructing a NIR quantitative model using a spectral library

The production process of amoxicillin capsules can be summarized as follows: granules are mixed with excipients after dry granulation of API and sieving; the mixed granules are then placed into capsules. The content of mixed intermediate granules of amoxicillin capsules ranged from 80.0 to 84.0%. The water content ranged from 12.1 to 13.0%. Mixed intermediate granules had only slight variability, so their NIR spectra were not suitable for a calibration set. We tried to set up NIR quantitative models for analyzing the content of amoxicillin and water in mixed intermediate granules of amoxicillin capsules using a spectral library of amoxicillin and potassium clavulanate ODFs because their spectra were similar.

The r_T_ values of 211 samples were calculated according to the definition of r_T_. The median value of r_T_ of sample spectra was 91.33%. The maximum and minimum r_T_ values were 99.29 and 88.98%, respectively. About 40 calibration spectra were selected from the spectral library. The difference in the adjacent spectra was 1.0–1.5%. The NIR model for amoxicillin was optimized by adding 16 spectra of mixed intermediate granules to the calibration set. The 13 spectra of mixed intermediate granules were added to the calibration set of the model for water content. Then, NIR quantitative models for the content of amoxicillin and water were constructed (Table 6). The prediction difference between the two models was small, so they could be used to analyze the content of amoxicillin and water of mixed granules rapidly during production.

**Table 6:** Parameters and prediction difference of models for the amoxicillin capsule constructed using a spectral library.

**Parameter**	**Amoxicillin Model**		**Water Model**	

	**Training set**	**Test set**	**Training set**	**Test set**
Number of samples	38	13	42	11
Content range (%, mg/mg)	7.97–84.40	8.05–84.31	1.42–12.80	1.89–12.60
Wavenumber range (cm^−1^)	7702.5–7124, 6549.3–5970.7, 4825.2–4246.6		9955.1–8848.1, 7752.7–7197.3	
Preprocessing method	1st derivative + vector normalization		1st derivative + vector normalization	
Rank	5		5	
*R*^2^ (%)	99.91		99.38	
RMSEP (%)	0.854		0.256	
Samples whose prediction difference is >5%	0% (0/211)		0% (0/211)	
Samples whose prediction difference is <1%	41.2% (87/211)		65.4% (138/211)	
Average prediction difference	0.8%		0.4%	

## Conclusions

A NIR spectral library of amoxicillin and potassium clavulanate ODFs was established using a universal model. The similarity between NIR spectra was represented by the correlation coefficient r_T_. About 30–50 calibration spectra were selected from the spectral library according to the median r_T_ value to construct the NIR quantitative model. The difference in r_T_ values between adjacent calibration spectra was about 1.0–1.5%. Compared with conventional modeling, this general method using a spectral library could be used to resolve sample-collection problems. This method requires calibration samples with an appropriate concentration range over a short time for PAT control. Furthermore, the quantitative models were more specific than models constructed by conventional methods. The proposed method offers a new and effective approach to solve the sample-selection problem in PAT modeling.

## Author contributions

All authors listed have made a substantial, direct and intellectual contribution to the work, and approved it for publication.

### Conflict of interest statement

The authors declare that the research was conducted in the absence of any commercial or financial relationships that could be construed as a potential conflict of interest.

## Footnotes

^1^Chinese Pharmacopeia 2015th Volume IV.103–104.
